# Lipopolysaccharide (LPS) Accumulates in Neocortical Neurons of Alzheimer’s Disease (AD) Brain and Impairs Transcription in Human Neuronal-Glial Primary Co-cultures

**DOI:** 10.3389/fnagi.2017.00407

**Published:** 2017-12-12

**Authors:** Yuhai Zhao, Lin Cong, Walter J. Lukiw

**Affiliations:** ^1^Neuroscience Center, Louisiana State University School of Medicine, Louisiana State University Health Sciences Center, New Orleans, LA, United States; ^2^Departments of Anatomy and Cell Biology, Louisiana State University School of Medicine, Louisiana State University Health Sciences Center, New Orleans, LA, United States; ^3^Department of Neurology, Shengjing Hospital, China Medical University, Shenyang, China; ^4^Department of Neurology, Louisiana State University School of Medicine, Louisiana State University Health Sciences Center, New Orleans, LA, United States; ^5^Department of Ophthalmology, Louisiana State University School of Medicine, Louisiana State University Health Sciences Center, New Orleans, LA, United States

**Keywords:** Alzheimer’s disease (AD), inflammatory degeneration, lipopolysaccharide (LPS), RNA Pol II transcription, run-on gene transcription

## Abstract

Several independent laboratories have recently reported the detection of bacterial nucleic acid sequences or bacterial-derived neurotoxins, such as highly inflammatory lipopolysaccharide (LPS), within Alzheimer’s disease (AD) affected brain tissues. Whether these bacterial neurotoxins originate from the gastrointestinal (GI) tract microbiome, a possible brain microbiome or some dormant pathological microbiome is currently not well understood. Previous studies indicate that the co-localization of pro-inflammatory LPS with AD-affected brain cell nuclei suggests that there may be a contribution of this neurotoxin to genotoxic events that support inflammatory neurodegeneration and failure in homeostatic gene expression. In this report we provide evidence that in sporadic AD, LPS progressively accumulates in neuronal parenchyma and appears to preferentially associate with the periphery of neuronal nuclei. Run-on transcription studies utilizing [α-^32^P]-uridine triphosphate incorporation into newly synthesized total RNA further indicates that human neuronal-glial (HNG) cells in primary co-culture incubated with LPS exhibit significantly reduced output of DNA transcription products. These studies suggest that in AD LPS may impair the efficient readout of neuronal genetic information normally required for the homeostatic operation of brain cell function and may contribute to a progressive disruption in the read-out of genetic information.

## Introduction—The Human GI Tract Microbiome

The human gastrointestinal (GI) tract microbiome is comprised of a complex and dynamic community of microbiota consisting predominantly of bacteria with various species of fungi, protozoa, viruses and other microorganisms making up the balance (Lukiw, 2016a,b; Pistollato et al., 2016; Zhan et al., 2016; Jiang Q. et al., 2017; Sherwin et al., 2017; Westfall et al., 2017). The number of GI tract microbial genes from 1000 different species of anaerobic or facultative anaerobic bacteria vastly outnumber human genes by at least one hundred to one (Foster et al., 2016; Lukiw, 2016a,b; Zhan et al., 2016; Jiang Q. et al., 2017; McManus and Heneka, 2017; Zhao et al., 2017a,b). There is a growing appreciation of the critical role that GI tract microbes play in health, aging and disease including their important roles in coordinating metabolic-, nutritive- and homeostatic-functions, and in their functional disruption in chronic diseases such as anxiety, autoimmune-disease, diabetes, metabolic-syndrome, obesity and stress-induced and progressive inflammatory neurodegenerative and neuropsychiatric diseases that include Alzheimer’s disease (AD). Microbes such as *Bacteroides fragilis* (*B. fragilis*) and *Escherichia coli* (*E. coli*), abundant Gram-negative bacilli of the human GI-tract microbiome, appear to accomplish these critical regulatory roles through the stress-induced secretion of a complex mixture of bacterial amyloids, endotoxins and exotoxins, small non-coding “microRNA-like” RNAs and lipopolysaccharide (LPS). Recently work from several independent groups has described the presence of bacterial nucleic acid sequences or bacterial-derived neurotoxins such as highly pro-inflammatory LPS associated with neuronal parenchyma and in particular the neuronal nuclei of the AD-affected brain (Bhattacharjee and Lukiw, 2013; Clark and Vissel, 2015; Foster et al., 2016; Bagyinszky et al., 2017; Bloch et al., 2017; McManus and Heneka, 2017; Zhao et al., 2017a,b).

In these experiments, we further investigated the association of LPS with sporadic AD and age-matched control hippocampus after the discovery of a strong association of LPS with neuronal cells and with the periphery of neuronal nuclei in AD brain (Bhattacharjee and Lukiw, 2013; Bagyinszky et al., 2017; Zhao et al., 2017a,b). Run-on transcription studies of human neuronal-glial (HNG) cells in primary culture using an extremely sensitive endogenous RNA Pol II activity driven incorporation of [α-^32^P]-uridine triphosphate (10^8^ dpm/ml) into newly synthesized total RNA indicated that nanomolar concentrations of LPS strongly inhibit neuronal nuclei transcriptional output. This may contribute in part to the generalized down-regulation of gene expression for transcription factors and synaptic and neurotrophic markers as is widely observed in sporadic AD brain (Colangelo et al., 2002; Ginsberg et al., 2012; Garcia-Esparcia et al., 2017; Itoh and Voskuhl, 2017).

## Materials and Methods

### Human Neuronal-Glial (HNG) Cells in Primary Co-culture

Culture of human neuronal-glia (HNG) primary cells, cryopreserved at passage one were obtained from commercial sources and cultured according to supplier’s instructions (Lonza PT-2599, Lonza Cell Systems, Allendale, NJ, USA or Cell Systems, ACBRI 376, Kirkland, WA, USA). HNG cells tested negative for HIV-1, HBV, HCV, mycoplasma, bacteria, yeast and fungi at source, have been extensively used for studies on brain gene expression, and demonstrate particular neuronal and astroglial cell markers including neuron-specific β-tubulin III (βtubIII; red staining) and astroglial-specific glial fibrillary acidic protein (GFAP; green staining). Briefly, HNG cells were maintained as free-floating aggregates (neurospheres) in 75 cm^2^ uncoated plastic flask in neural progenitor maintenance media (Lonza CC-3209) supplemented with human recombinant fibroblast growth factor (rhFGF) and epidermal growth factor [rhEGF]) and neural survival factor-1 [NSF-1] (Lonza CC-4242) and gentamicin/amphotericin-B (Lonza GA-1000). Differentiation was induced by plating neurospheres onto 8-well glass chamber-slides pre-coated with poly-L-ornithine (an amino acid polymer used as substratum to improve neuronal adhesion). The differentiation media (Lonza CC-4242) was free of growth factors but contained NSF and gentamicin/amphotericin-B, 25 ng/ml of brain-derived neurotrophic factor (BDNF) and 1% of fetal bovine serum (FBS). Upon deprivation of growth factors neurospheres started to attach to bottom of wells and migrate out to form a co-culture of neurons and glial cells (HNG). Experimental treatment started at 2 weeks after induction of differentiation. The cells were kept at 37°C in a humidified 5% CO_2_ atmosphere incubator at all times. HNG cells initially contained about 5 × 10^5^ cells/ml volume and were cultured to ~70% confluency in HNG cell medium as described in detail (Cui et al., 2010; Bhattacharjee and Lukiw, 2013; Zhao et al., 2014, 2017a; Foster et al., 2016; Lukiw, 2016a,b; Pistollato et al., 2016; Zhan et al., 2016; Bagyinszky et al., 2017; Jiang Q. et al., 2017; Li and Yu, 2017; McManus and Heneka, 2017; Sherwin et al., 2017; Westfall et al., 2017; http://www.mikroskop.com.pl/pdf/LSM700_1.pdf).

### Human Brain Tissues, Antibodies and Immunohistochemistry

Age matched female control (*N* = 12; age 85.8 ± 2.1 years and PMI 3.6 ± 1.5 h) and AD (*N* = 15; age 87.7 ± 2.5 years and PMI 3.8 ± 1.2 h) human superior temporal lobe neocortical tissues (Brodmann A22) were obtained from UC-Irvine Brain Bank, the University of Maryland and archived material at the Louisiana State University Neuroscience Center. A total of 24 age-, gender (all females) and PMI-matched control and AD brains were examined for LPS immunostaining and/or run-on transcription analysis. For LPS immunocytochemistry human brain tissue samples were embedded in OCT and frozen at −80°C; brain sections (10 μm) were cut on a Shandon cryotome (Waltham, MA, USA). After an initial fixation with 4% paraformaldehyde for 20 min, sections were then incubated in primary antibodies (1:1000; 1× PBS with 2% BSA, 2% goat or donkey serum and 0.1% TX-100) overnight at 4°C, washed with PBS and then incubated with Alexa Fluor-conjugated species-specific secondary antibodies (ThermoFisher Scientific, Waltham, MA, USA) for 3 h at RT. Sections were counter-stained with DAPI for nuclei, followed by quenching with Autofluorescence Eliminator Reagent (Millipore Cat # 2160; Zhan et al., 2016), mounted on glass slides, cover-slipped with Fluoromount-G (ThermoFisher Scientific) and imaged using a Zeiss LSM 700 Confocal Laser Scanning microscope system (Carl Zeiss Microscopy, Thornwood, NY, USA; Bagyinszky et al., 2017; Zhao et al., 2017a; http://www.mikroskop.com.pl/pdf/LSM700_1.pdf).

### Immunofluorescence Protocol

HNG cells cultured on 8-well Chamber Slide (BD Biosciences, San Jose, CA, USA) were fixed with 4% paraformaldehyde, then permeabilized and blocked with 0.125% Triton X-100 and 2% normal goat serum in PBS at RT for 1 h. Cells were incubated overnight at 4°C with antibodies for β-tubulin III (for neurons; Sigma T8578, Sigma-Aldrich St. Louis, MO, USA) and GFAP (for astrocytes; Sigma G9629). Later cells were washed for three times with PBS and then incubated for 3 h at room temperature with secondary antibodies conjugated with cy3 or FITC fluorescein (Thermofisher A21422 and A11008; ThermoFisher Scientific, Waltham, MA, USA). After being washed and dried, slides were applied with mounting medium containing DAPI (1:10,000; Vector Laboratories, Burlingame, CA, USA) and observed under Zeiss Axioplan Inverted Deconvolution Fluorescent Microscope (63× oil immersion lens; Carl Zeiss, Oberkochen, Germany). Positively stained cells were quantified manually by a using manual counter function of ImageJ software. Negative control with quenching was performed and the data are attached in Supplementary Figure S2; we performed quantification on LPS as percentage of neuronal area. Antibodies used: mouse anti-*E. coli* LPS (Abcam, Cat# ab35654; Abcam, Cambridge, MA, USA) and rabbit anti-NeuN (Cell Signaling, Cat# 24307), rabbit anti-GFAP (Sigma-Aldrich, Cat# G4564; Lukiw et al., 1998; Cui et al., 2010; Zhao et al., 2014; Lukiw, 2016a,b; Zhan et al., 2016). LPS antibody specificity and validation was confirmed using Western immunoblot analysis (Figure 1 in Zhao et al., 2017a) which corresponded to the product specifications (http://www.abcam.com/e-coli-lps-antibody-ab211144.html) and an antibody neutralization/LPS quenching control assay (Supplementary Figure S2; Skliris et al., 2009; Bordeaux et al., 2010). To ascertain the association of LPS with neuronal cells confocal images of LPS and NeuN staining were imported into ImageJ (https://imagej.nih.gov/ij/); RGB images were first converted into images of separate channels (red for LPS and green for NeuN). A co-localization finder plugin was run to generate images of co-localization of both channels; each co-localization image was converted into an 8-bit image and inverted. Global thresholding was used and the cutoff value was adjusted to the point that only highlighted co-localized particles are black on the image against a white background. Particle analysis was then performed to calculate the area size of the co-localization; this value was then divided by the area size of the NeuN staining as the percentage of cell area.

### Run-on Transcription and Total [α-^32^P]-UTP–labeled RNA-driven Hybridizations

Run-on transcription using endogenous RNA polymerase II (RNA Pol II) incorporation of [α-^32^P]-UTP into newly synthesized RNA and total control or AD messenger RNA (mRNA) has been previously described by our labs and others in considerable detail (Lukiw et al., 1998; Ricicová and Palková, 2003; Cui et al., 2005; Smale, 2009; http://www.genomics.agilent.com/en/Bioanalyzer-DNA-RNA-Kits/RNA-Analysis-Kits/?cid=AG-PT-105&tabId=AG-PR-1172). Briefly, total messenger RNA (mRNA) was isolated using Trizol reagent (Gibco-BRL, Gaithersburg, MD, USA); spectral quality, quantity, and purity of total mRNA was determined by scanning RNA in sterile RNase-free water from 220 nm to 320 nm using a Beckman (Fullerton, CA, USA) DU-65 spectrophotometer and an Agilent Bioanalyzer using RNA 6000 Nano Assay (sensitivity ~20 ng μl^−1^; 18–20). Dot blots containing 0.5, 1.0, 2.0 and 5.0-μg DNA probes for the human-specific Alu repetitive element, the neuron-specific NFL chain gene and the astroglial-specific glial fibrillary acidic protein (GFAP) DNA on 4 × 8 cm HyBond N+ membrane panels (Amersham) were probed with total [α-^32^P]-UTP radiolabeled RNA (10^8^ dpm/mL) using a Bio-Rad (Hercules, CA, USA) Bio-dot SF blot system. Total [α-^32^P]-UTP labeled total RNA was hybridized 3 h to the DNA immobilized on the dot blot panels using 5 mL of ExpressHyb hybridization solution (Clontech, Palo Alto, CA, USA). Panels were washed to moderate-to-high stringency using 20× SSC (3 M NaCl, 0.3 M Na-Citrate) and 0.1% SDS at 50°C according to the manufacturer’s protocol (Clontech). Autoradiograms were generated using either Kodak Biomax MS film or by exposing membranes to phosphorimager storage screens and analyzing the resulting signals using a Bio-Rad GS250 molecular imager or a Fuji FLA2000 Bio-Imaging Analyzer (FujiFilm Corporation, Tokyo, Japan). Relative intensities of Alu, NFL, or GFAP signals were quantitated using the data acquisition and statistical analysis packages provided with each instrument. For further complete description please refer to a previous publication from our laboratory and collaborators (Lukiw et al., 1998; Ricicová and Palková, 2003; Cui et al., 2005; Smale, 2009; http://www.genomics.agilent.com/en/Bioanalyzer-DNA-RNA-Kits/RNA-Analysis-Kits/?cid=AG-PT-105&tabId=AG-PR-1172).

### Statistical Analysis, Integrated Bioinformatics Analysis and Data Interpretation

For Alu, NFL and GFAP mRNA abundance analysis all statistical procedures were analyzed using (*p*, analysis of variance (ANOVA)) a two-way factorial analysis of variance using algorithms and/or procedures in the SAS language (Statistical Analysis Institute, Cary, NC, USA) and as previously described (Cui et al., 2010; Zhao et al., 2011; Clement et al., 2016; Dendooven and Luisi, 2017). In the results *p*-values of less than 0.05 (ANOVA) were considered to be statistically significant. All Alu, NFL and GFAP mRNA abundance data were collected and analyzed using Excel 2016 (Office 365) algorithms (Microsoft Corporation, Redmond WA, USA); all figures were generated using Adobe Illustrator CC 2015 and Photoshop CC version14.0 (Adobe Corporation, San Jose, CA, USA).

## Results

**Figure 1:**
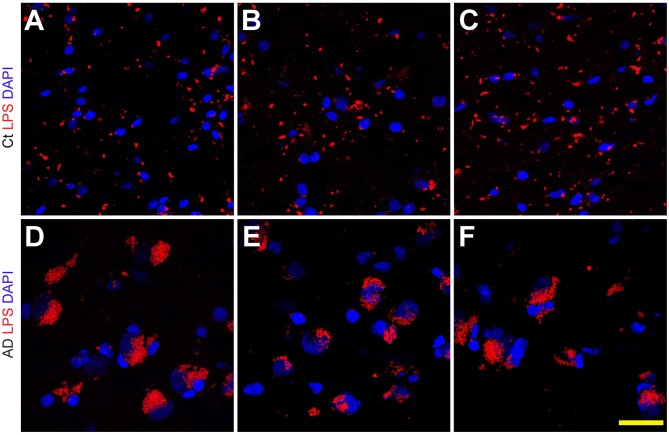
Lipopolysaccharide (LPS) staining in the human superior temporal lobe neocortex (Brodmann A22) in control and Alzheimer’s disease (AD) brain; LPS (red stain; *λ*_max_ = 690 nm) and DAPI (blue stain; *λ*_max_ = 470 nm) staining of three control (Ct) (panels **A–C**) and three age-matched AD (panels **D–F**) temporal lobe neocortical tissues; the size of all microscope fields in this photo are equal; interestingly the slightly larger size of some DAPI-stained AD nuclei (blue) in panels **(D–F)** has been previously described in neuropathological studies of multiple anatomical regions in AD brain; the reason for this nuclear hypertrophy and plasticity in AD neuronal nuclei not entirely clear, but may be a “compensatory” mechanism requiring a more “expanded” euchromatin and increased transcriptional activity for some neurons that are attempting to repair neuronal and synaptic damage (Iacono et al., 2008); all panels magnification 63×; scale bar = 20 μm.

**Figure 2:**
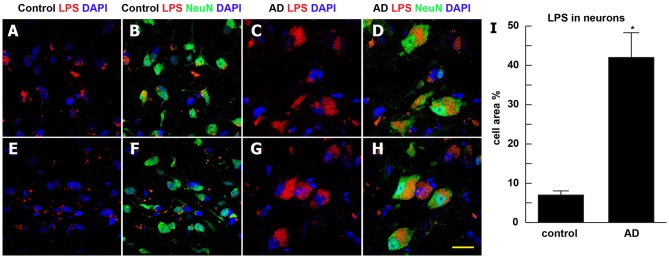
Association of LPS with the periphery of neuronal nuclei in AD neocortex—LPS (red stain; *λ*_max_ = 690 nm), DAPI (blue stain; *λ*_max_ = 470 nm) and NeuN (green stain; *λ*_max_ = 520 nm) staining of control and age-matched human superior temporal lobe AD neocortex (Brodmann A22); note that in the right-most AD panels **(C–G,D,H)** about 90% of all LPS signals were associated with NeuN (green-staining; neuronal) and DAPI (blue staining) nuclei; panels **(A,B,E,F)** are from control neocortex; panels **(C,D,G,H)** are from AD neocortex: quantitative analysis of LPS association with neuronal cells in bar graph format (panel **I**); LPS staining (red) was quantified as average percentage of neuronal area associated with neuronal cells (green); LPS staining (red) was subjected to co-localization analysis with the neuronal marker NeuN (green) and/or nuclear marker (blue); the highlighted co-localized area was then quantified as a percentage of neuronal area in the image; the analysis was performed by using NIH ImageJ software (see text for further details); data are presented as one mean ± one standard deviation (SD); *p < 0.05 vs. control; for all panels magnification 63×; scale bar = 20 μm.

Staining of human temporal lobe neocortical sections from control and age- and gender-matched AD brains with anti-LPS fluorescent antibodies showed detectable signals in both cases, however the control LPS signals (Figures 1A–C) were more disperse and punctate while the AD LPS signals (Figures 1D–F) were more self-associating, globular and abundant, and were almost always associated with NeuN- and DAPI-staining neuronal nuclei (Supplementary Figure S1; see Figure 2). AD LPS signal yields in neuronal cells averaged at least 7-fold or greater than controls in this brain region. In order to investigate how LPS may be associating with neuronal nuclei, a series of temporal lobe neocortical sections from control and age-matched AD brains were stained with LPS and a DAPI nuclear stain (control Figures 2A,E; AD Figures 2C,G) as well as the neuron-specific stain NeuN (control Figures 2B,F; AD Figures 2D,H); the results of two control brains (both female; mean age 85.5 ± 3.1 years and PMI 3 h or less; Figures 2A,B,E,F) and two AD brains (both female age 86.5 ± 2.5 years and PMI 3 h or less; Figures 2C,D,G,H) are representative of assays on multiple brains (*N* = 12). In AD LPS accumulation was associated with the nuclei of neurons; in moderate-to-late-stage AD some neuronal nuclei were almost completely surrounded by LPS (see Figures 2D,H; Yang et al., 2008; Gorman, 2008; Jellinger, 2010; unpublished observations).

**Figure 3:**
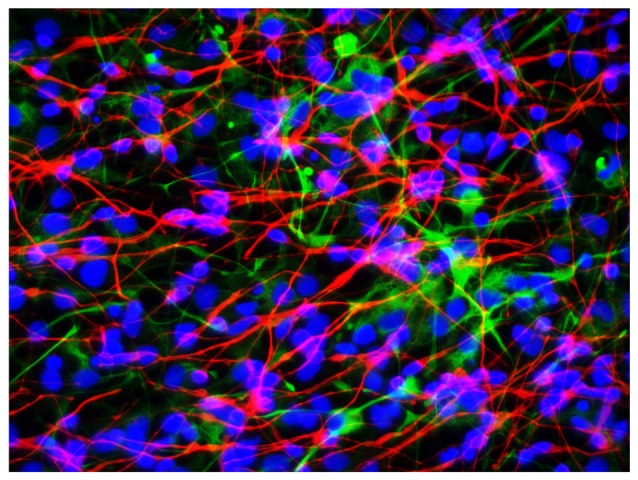
Primary human neuronal-glial (HNG) cells after ~2 weeks in primary co-culture; the cell density is approximately 75% neurons and 25% astroglia at ~60% confluency; human primary neuronal and glial “support” cell co-cultures are utilized, because human neuronal cells do not culture well by themselves (Cui et al., 2010); neuronal cells are stained with neuron-specific β-tubulin (red; *λ*_max_ = 690 nm), glial cells are stained with glial-specific glial fibrillary acidic protein (GFAP; green; *λ*_max_ = 525 nm), and nuclei are stained with DAPI/Hoechst 33258 stain (blue; *λ*_max_ = 470 nm); photo magnification 30×; scale bar = 50 μm.

We next quantified the effects of LPS (at 0, 50, 100, 500 and 1000 nM with exposure for 36 h) on transcriptional capability in primary HNG cells after ~2.5 weeks in primary co-culture (the HNG cell density is approximately 75% neurons and 25% astroglia at ~60% confluency) using run-on gene transcription (Lukiw et al., 1998; Ricicová and Palková, 2003; Cui et al., 2005; Smale, 2009; Figure 3). Three different types of DNA transcripts were quantified, the Alu repetitive element RNA as an index of general RNA polymerase type II (RNA Pol II) transcriptional activity, the NFL chain mRNA which is an abundant and essential neuron-specific cytoskeletal intermediate filament responsible in part for the cytoarchitecture of the neuron, and glial fibrillary acidic protein (GFAP), an abundant astroglial-specific intermediate filament important in maintaining the 3-dimensional shape of astroglial cells (Table 1). Interestingly, at just 100 nM ambient LPS the yield of newly synthesized Alu, NFL and GFAP transcription products was reduced to 41, 16 and 81 percent of controls, indicating that LPS may have a repressive effect on global gene activity and a more focused effect on neuronal transcript output vs. that of astroglial cells (Table 1).

**Table 1:** Run-on transcription—impairment of neuronal transcriptional output by lipopolysaccharide (LPS).

LPS (nM)	*n*	Alu	NFL	GFAP
0 (control)	8	100	100	100
50	10	54 ± 9.3	27 ± 8.7	86 ± 9.1
100	5	41 ± 10.3	16 ± 9.8	81 ± 11.2
500	5	36 ± 11.3	15 ± 11.3	72 ± 11.3
1000	5	35 ± 11.3	12 ± 11.3	72 ± 11.3

*HNG cells in primary culture (Figure 3) were used to program run-on gene transcription, a highly informative and sensitive method for monitoring the mRNA generating capacity of any cell system (Lukiw et al., 1998; Ricicová and Palková, 2003; Cui et al., 2005; Smale, 2009; http://www.genomics.agilent.com/en/Bioanalyzer-DNA-RNA-Kits/RNA-Analysis-Kits/?cid=AG-PT-105&tabId=AG-PR-1172). Effect of various doses of LPS (nM) on *in vitro* RNAP II [mRNA transcripts coding for heterogeneous nuclear RNA (Alu) or for protein neurofilament light (NFL, GFAP)] activities in isolated human neocortical nuclei using Alu, NFL, or GFAP probes and expressed as a mean of control values ± one standard deviation (SD); n, number of individual run-on transcription experiments; Alu = Alu repetitive element; NFL, neurofilament light chain (neuron-specific marker); GFAP, glial fibrillary acidic protein (glial specific marker); LPS association with neuronal nuclei may block mRNA trafficking through nuclear pores and/or provide a biophysical barrier to restrict mRNA exit from the nucleus generating a mRNA-mediated down-regulation in gene expression as is widely observed in AD brain (Colangelo et al., 2002; Ginsberg et al., 2012; Garcia-Esparcia et al., 2017; Itoh and Voskuhl, 2017)*.

## Discussion and Conclusion

### Bacterial-derived Secretory Products and Prokaryotic Nucleic Acids in AD-affected Brain Cells

It has only recently become appreciated that in *Homo sapiens* microbial genes outnumber human genes by about 100 to 1, and the potential impact of bacterial secretory products and bacterial genetics and on human health, aging and disease may have been vastly underestimated (Bhattacharjee and Lukiw, 2013; Hill and Lukiw, 2015; Zhao and Lukiw, 2015; Lukiw, 2016a,b; Zhan et al., 2016; Bagyinszky et al., 2017; Emery et al., 2017; Jiang Q. et al., 2017; Zhao et al., 2017a,b). The supposition of a *“privileged immunological status for the mammalian CNS”* has also been recently questioned in neuropathological and innate-immune system genetic studies of AD and murine amyloid-overexpressing transgenic models for AD, particularly in terms of inflammatory neurodegeneration. Both microbial-derived nucleic acid sequences and/or noxious exudates representative of GI-tract Gram-negative bacteria are showing up in anatomical regions of the CNS involved in inflammatory and neuro-immune disruptions that strongly associate with the AD process (Bhattacharjee and Lukiw, 2013; Zhao et al., 2014, 2017a,b; Hill and Lukiw, 2015; Zhao and Lukiw, 2015; Foster et al., 2016; Lukiw, 2016a,b; Zhan et al., 2016; Bagyinszky et al., 2017; Emery et al., 2017; Jiang Q. et al., 2017). To cite several very recent examples: (i) using immunological methods Zhao et al. (2015, 2017a) discovered LPS in very short post-mortem interval AD hippocampus to levels 30-fold or greater than age-matched controls; (ii) using immunocytochemistry Sharp’s group found *E. coli* K99 pili protein and LPS levels significantly greater in AD compared to control brains, finding that in AD LPS co-localized with Aβ1–40/42-positive amyloid plaques and around cerebral vessels (Zhan et al., 2016); (iii) 16S rRNA next generation sequencing analysis identified multiple bacterial nucleic acids in AD brains (Emery et al., 2017); and (iv) Zhao et al. (2017a) found an specific enrichment of LPS specifically associated with the neuronal nuclear membrane in AD brain. All of these findings suggest that LPS and other bacterial-derived amyloids and neurotoxins are localized to the same anatomical regions involved in AD-type neuropathology and may be a significant initiator or progressive contributor to inflammatory degeneration and/or an altered innate-immune response in the AD CNS (Figures 1, 2).

### Noxious Exudates of the Human GI-Tract Microbiome—LPS Structure and AD-relevant Interactions

*Bacteroides fragilis* (*B. fragilis*) and *Escherichia coli* (*E. coli*), abundant Gram-negative bacilli of the human middle and lower GI-tract microbiome, have potential to secrete an extraordinarily complex mixture of pathogenic bacterial amyloids, exotoxins, small non-coding RNAs (sncRNAs) and LPS (Hill and Lukiw, 2015; Zhao and Lukiw, 2015; Köhler et al., 2016; Lukiw, 2016a,b; Bergman et al., 2016; Jiang Q. et al., 2017; VanItallie, 2017; Zhao et al., 2017a; unpublished observation). As major anaerobic Gram-negative bacilli of the human middle and lower GI-tract, respectively; the *B. fragilis* exotoxin (BFT) fragilysin is one of the most potent pro-inflammatory molecules known (Zhao and Lukiw, 2015; Lukiw, 2016a,b; Nitzan et al., 2017; Zhao et al., 2017a; http://www.mikroskop.com.pl/pdf/LSM700_1.pdf); these intensely pro-inflammatory LPS species may be able to “leak” through at least two major biophysical barriers—the GI-tract barrier and the blood–brain barrier—to ultimately access brain compartments (Köhler et al., 2016; Varatharaj and Galea, 2017; Zhao et al., 2017b). Increased GI tract and blood-brain barrier permeability induced by microbiota dysbiosis may mediate or affect AD pathogenesis and other neurodegenerative disorders, especially those associated with aging. As heat stable 10–20 kDa lipid endotoxins covalently modified with polysaccharides of the outer membrane of Gram-negative bacteria, LPS monomers generally consist of three parts: (i) a repetitive hydrophilic glycan polymer known as the “O”-lipid specific to the bacterial serotype; (ii) a hydrophilic core polysaccharide component necessary for activation of the pro-inflammatory transcription factor NF-κB and immune-related microRNA-146a; and (iii) a hydrophobic, toxic lipid “A” consisting of two glucosamine groups with attached fatty acids, often containing one phosphate on each glucosamine (http://www.sigmaaldrich.com/technical-documents/articles/biology/glycobiology/lipopolysaccharides, Pogue et al., 2009; Zhao et al., 2017a). LPS typically shield Gram-negative bacilli against the action of bile salts and lipophilic antibiotics thus playing a role in host–pathogen immune-evasion strategies useful to bacterial survival while eliciting intense immune and pro-inflammatory responses within the host (Jiang C. et al., 2017; Torres-Martínez and Ruiz-Vázquez, 2017; http://www.sigmaaldrich.com/technical-documents/articles/biology/glycobiology/lipopolysaccharides). Interestingly, secreted LPS, along with proteolytic endotoxins, amyloids and sncRNAs, over time can aggregate into insoluble fibrous lipoprotein lesions that accumulate in brain parenchyma and associate with the progressive and lethal degenerative neuropathology of the human CNS that includes AD and prion disease (Hill and Lukiw, 2015; Bhattacharjee and Lukiw, 2013; Foster et al., 2016) Thus, LPS, as the major molecular component of the outer membrane of Gram-negative bacteria, normally: (i) may serve as a physical barrier providing the bacteria evasion from the anti-microbial actions of the host; (ii) may be recognized by the immune system as a marker for the detection of bacterial pathogen invasion and responsible for the development of inflammatory responses; and (iii) within the CNS is perhaps the most potent stimulator and trigger of an inflammatory response known (Köhler et al., 2016; Lukiw, 2016a,b; McManus and Heneka, 2017; http://www.sigmaaldrich.com/technical-documents/articles/biology/glycobiology/lipopolysaccharides.html).

Biophysical or biochemical parameters regulating the natural affinity of LPS for the nuclear region of neuronal nuclei are not well understood. In chronic and fatal neurofibrillary degenerative pathologies that include AD and prion disease, LPS has been previously shown to strongly interact with a non-conventional, non-nuclear isoform of histone H1, the major neuronal membrane-associated LPS-binding protein in the brain and a putative cell surface receptor that: (i) may be part of the neuronal or nuclear cytoskeleton; and (ii) may be associated with nucleoproteins which comprise nuclear pore ring structures (Bolton and Perry, 1997; Duce et al., 2006; Figure 2). Linker histone H1, with a primary role of binding DNA that enters and exits the nucleosome to condense chromatin into a more “heterochromatic” or “quiescent” state, also strongly interacts with Congo red staining amyloid fibrils (Duce et al., 2006). Of related interest is that LPS activates toll-like receptors (TLRs), and more specifically TLR2 and TLR4, membrane-spanning protein receptors expressed in microglial cells of the innate-immune system, which recognize common damage- or pathogen-associated molecular-patterns (DAMPS or PAMPs; Kigerl et al., 2014; Lukiw, 2016a,b; Mathur et al., 2017). There is evidence that LPS-TLR interactions trigger inflammation, phagocytosis, and innate-immune defense responses that directly induce the development of CNS pathology, but how LPS-nuclear membrane attraction parameters change with aging and in disease are not well understood (Pardon, 2015; Jiang C. et al., 2017; Li and Yu, 2017; http://www.sigmaaldrich.com/technical-documents/articles/biology/glycobiology/lipopolysaccharides).

### LPS Represses Transcriptional Activity in Neurons

This current work also quantified the abundance of Alu repetitive RNA, NFL (neuron-specific) mRNA and (glial-specific) GFAP mRNA (Figure 3 and Table 1) in control and LPS-treated HNG cells using run-on transcription (Lukiw et al., 1998; Ricicová and Palková, 2003; Cui et al., 2005; Smale, 2009). These three transcription products are reflective of RNA Pol II activity in brain cells with Alu abundance being significantly expressed in all brain cells, NFL being neuron-specific and GFAP representing glial-specific transcripts. Alu and NFL mRNA abundance were found to be reduced in the presence of LPS. Interestingly, multiple laboratories have shown previously Alu and NFL gene expression to be down-regulated in AD and in murine models of AD (Lukiw et al., 1990, 1992; McLachlan et al., 1991; Takano et al., 2013; Itoh and Voskuhl, 2017) although NFL levels appear to be increased in blood plasma in AD (Zhou et al., 2017). While the evidence presented here suggests that there may be preferential binding of LPS to the neuronal nuclear region in sporadic AD, a consequence of this may be an inability of mRNA to freely exit the nucleus resulting in a global repression of transcriptional output in neurons. Studies are underway to ascertain to which neuronal peripheral structures LPS is attracted, if LPS that has reached CNS compartments selectively down-regulates other neuron-specific microRNA and/or mRNA species, and how this may impact innate-immune or inflammatory signaling functions in the aging and AD-affected brain.

## Ethics Statement

All procedures and protocols were followed and human tissues handled in strict accordance with the Biosecurity and Institutional Biosafety Committee/Institutional Review Board (IBC/IRB) and ethical guidelines at the LSU Health Sciences Center, LA 70112 (IBC#12323; IRB#6774).

## Author Contributions

YZ, LC and WJL acquired human control and AD brain samples from multiple sources, cultured primary human neuronal-glial (HNG) cells, YZ and LC performed all immunocytochemistry analysis, WJL performed run-on gene transcription analysis; WJL coordinated data and wrote the article.

## Conflict of Interest Statement

The authors declare that the research was conducted in the absence of any commercial or financial relationships that could be construed as a potential conflict of interest.
