# Supplementary material for: Lipopolysaccharide (LPS) Accumulates in Neocortical Neurons of Alzheimer’s Disease (AD) Brain and Impairs Transcription in Human Neuronal-Glial Primary Co-cultures
Source: Front Aging Neurosci. 2017 Dec 12;9:407. doi: 10.3389/fnagi.2017.00407 (PMC5732913; doi:10.3389/fnagi.2017.00407)
Supplement: Supplementary file 1 [file Data_Sheet_1.docx]

**Supplementary file S1**

**
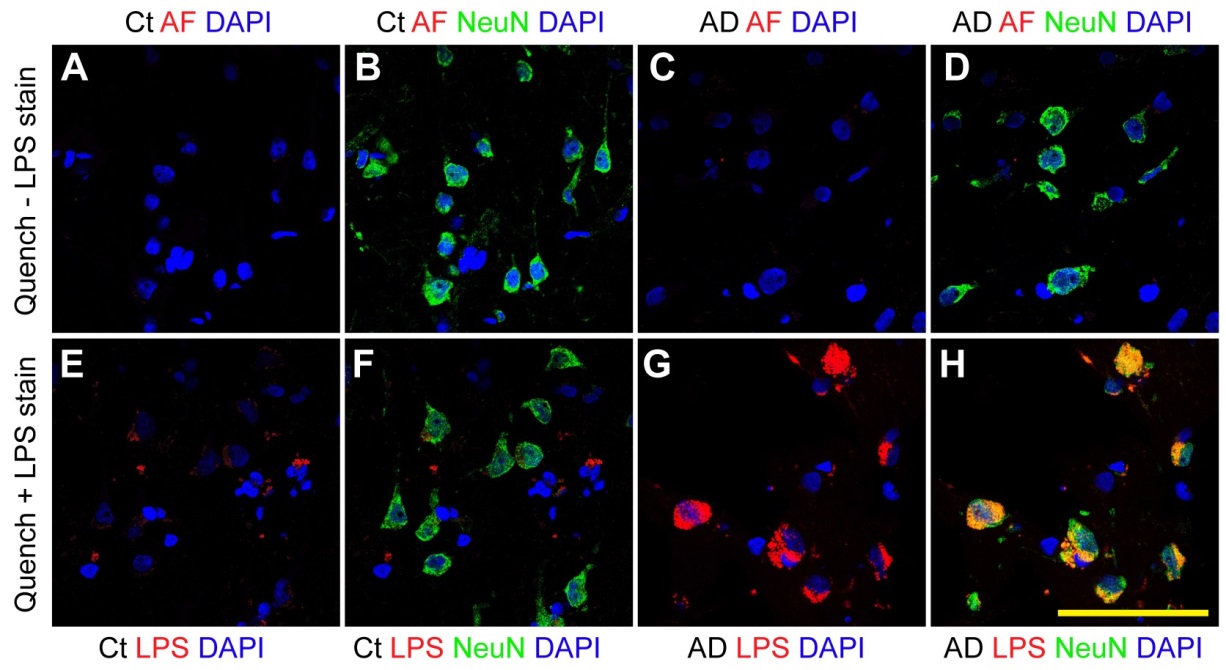
**

**Supplementary file S1 - Minimal auto-fluorescence in negative control of human brain LPS staining;** gender and age-matched control (Ct) and Alzheimer’s disease (AD) superior temporal lobe tissue subjected to two different immunofluorescent staining protocol quenching: without (quench – LPS staining, served as negative control) or with (quench + LPS staining). Minimal to no auto-fluorescence is observed in the negative control images; LPS (red stain; λ**_max_** = 690 nm), DAPI (blue stain; λ**_max_** = 470 nm) and NeuN (green stain; λ**_max_** = 520 nm); Ct = control; LPS=red stain for LPS; NeuN=stain for neuronal cells; DAPI = stain for nuclei; magnification 63x; scale bar = 50 um.

**Supplementary file S2**


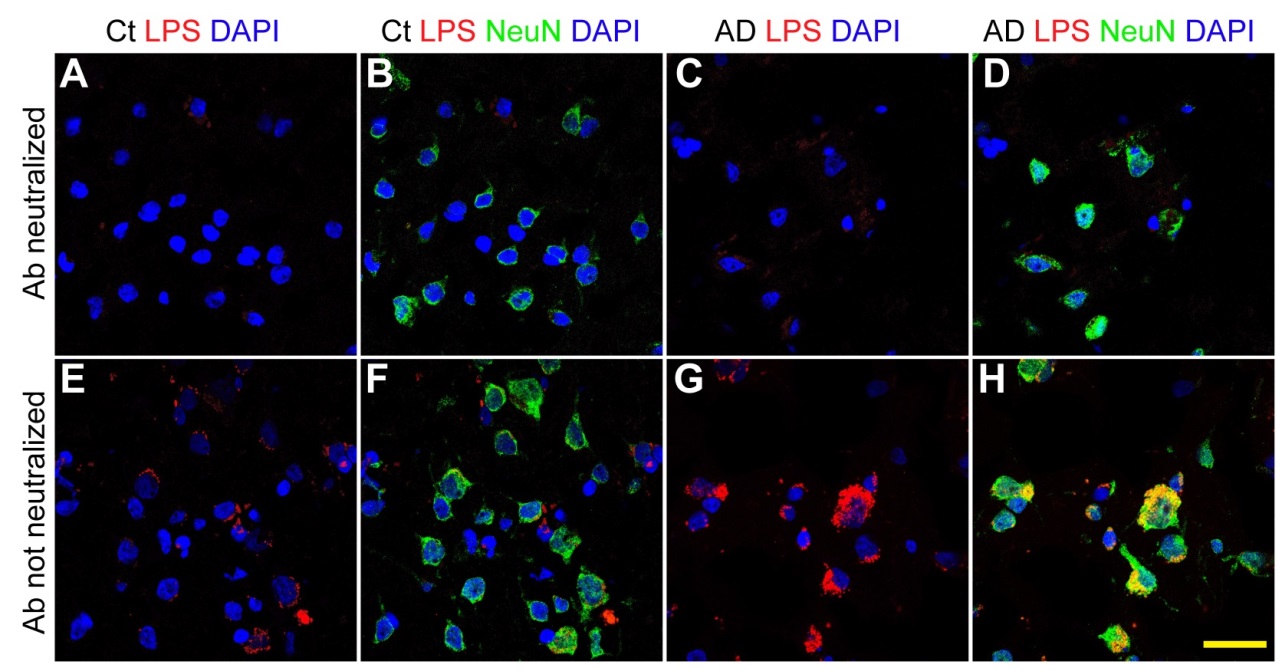


**Supplementary file S2 – Immuno-histochemical validation of LPS antibody;** Antibody validation conducted as previously reported (Skliris et al., 2009; Bordeaux et al., 2010; Zhao et al., 2017b). Anti- E. coli LPS monoclonal antibody (Abcam Cat# 35654; Abcam, Cambridge MA, USA) was either neutralized (immune-absorbed) with excessive amount of LPS (γ-irradiated, cell culture grade; Sigma-Aldrich Cat# L4391; Sigma-Aldrich, St. Louis MO, USA) or with a non-related immunogen TNF-α (recombinant, cell culture grade Sigma-Aldrich Cat# 8916) before being applied to brain sections of control and AD subjects for immunofluorescent staining. Pre-immuno-absorption with LPS resulted in negative staining of LPS (**A-D**) while incubation with non-related immunogen does not affect LPS staining (**E-H**); LPS (red stain; λ**_max_** = 690 nm), DAPI (blue stain; λ**_max_** = 470 nm) and NeuN (green stain; λ**_max_** = 520 nm); Ct = control; LPS=red stain for LPS; NeuN=stain specific for neuronal cells; DAPI = stain for nuclei; magnification 63x; scale bar = 20 µm.
